# Machine learning models for predicting unscheduled return visits to an emergency department: a scoping review

**DOI:** 10.1186/s12873-024-00939-6

**Published:** 2024-01-30

**Authors:** Yi-Chih Lee, Chip-Jin Ng, Chun-Chuan Hsu, Chien-Wei Cheng, Shou-Yen Chen

**Affiliations:** 1 Department of Emergency Medicine, Chang Gung Memorial Hospital and Chang Gung University, College of Medicine, Taoyuan City, 333 Taiwan; 2 Department of Emergency Medicine, Chang Gung Memorial Hospital, Keelung and Chang Gung University, College of Medicine, No. 5 Fushing St., Gueishan Shiang, Taoyuan City, 333 Taiwan

**Keywords:** Unscheduled return visit, Reattendance, Machine learning, Emergency department

## Abstract

**Background:**

Unscheduled return visits (URVs) to emergency departments (EDs) are used to assess the quality of care in EDs. Machine learning (ML) models can incorporate a wide range of complex predictors to identify high-risk patients and reduce errors to save time and cost. However, the accuracy and practicality of such models are questionable. This review compares the predictive power of multiple ML models and examines the effects of multiple research factors on these models’ performance in predicting URVs to EDs.

**Methods:**

We conducted the present scoping review by searching eight databases for data from 2010 to 2023. The criteria focused on eligible articles that used ML to predict ED return visits. The primary outcome was the predictive performances of the ML models, and results were analyzed on the basis of intervals of return visits, patient population, and research scale.

**Results:**

A total of 582 articles were identified through the database search, with 14 articles selected for detailed analysis. Logistic regression was the most widely used method; however, eXtreme Gradient Boosting generally exhibited superior performance. Variations in visit interval, target group, and research scale did not significantly affect the predictive power of the models.

**Conclusion:**

This is the first study to summarize the use of ML for predicting URVs in ED patients. The development of practical ML prediction models for ED URVs is feasible, but improving the accuracy of predicting ED URVs to beyond 0.75 remains a challenge. Including multiple data sources and dimensions is key for enabling ML models to achieve high accuracy; however, such inclusion could be challenging within a limited timeframe. The application of ML models for predicting ED URVs may improve patient safety and reduce medical costs by decreasing the frequency of URVs. Further research is necessary to explore the real-world efficacy of ML models.

## Introduction

Hospitals have been using unscheduled return visit (URV) rates and reattendance rates to assess the quality of care in their emergency departments (EDs) for many years. Higher URV rates not only increase health-care costs but also prolong wait times for patients who need immediate ED care. Studies have revealed that frequent visits to EDs significantly contribute to overcrowding in EDs, which can lead to delays in treatment and consequently higher mortality rates [1, 2]. Therefore, developing a predictive model for health-care systems is crucial for the adoption of early interventions to reduce ED revisits [3, 4].

Accurate predictive modeling is crucial for the development of interventions. URVs can be classified into illness-, doctor-, and patient-related returns; however, differentiating between these categories can be difficult [5]. Because of the multifaceted and complex nature of ED URVs a number of variables may affect URVs. Conventional statistical models are limited in their ability to identify high-risk patients because of these models’ reliance on preprogrammed rules derived from specific clinical predictors. By contrast, machine learning (ML) prediction models utilize nonparametric algorithms, which can incorporate a relatively comprehensive range of complex predictors while maintaining strong predictive performance [6, 7]. In addition, using ML-based methods can reduce errors, yield time and cost savings, and improve the quality of care services [8]. The use of ML models in predicting URVs to EDs has been investigated previously, and some studies have published their results [3, 9–21]. However, variations in methodology, patient population, research scale, and time interval have made determining the accuracy and practicality of using ML models to predict URVs to EDs difficult [22].

The present scoping review compared the predictive power of multiple ML models, assessed the proportion of methods used among the selected articles of each of these models, and examined the effects of multiple research factors on the performance of the models in predicting URVs. Additionally, we explored the clinical relevance of current ML models in predicting ED URVs.

## Materials and methods

### Information sources and search strategy

The protocol for this scoping review was developed on the basis of guidelines of the preferred reporting items for systematic review and meta-analysis (PRISMA) protocols statement [23] and was registered with the Open Science Framework [24]. The reporting of the present scoping review adheres to the PRISMA extension for scoping reviews [25]. We conducted a systematic search of eight databases (PubMed, ScienceDirect, the Global Health database, Embase, EconLit, Caim. Info, BDPS, and the Cochrane Library), using the following search terms of “Machine learning,” “Artificial intelligence,” “Emergency department,” “Emergency room,” “Predicting model,” “Predictive model,” “Unscheduled return,” “Unscheduled return visits,” “Reattendance,” and “Revisits.” Only studies published between January 2010 and February 2023 were selected to assess developments.

### Selection process and eligibility criteria

Inclusion and exclusion criteria were established to identify relevant articles for this study. Published studies of any study design were considered eligible for inclusion, whereas conference abstracts and grey literature such as unpublished research, policy statements, and government reports were excluded.

Selected articles were required to meet the following criteria: (i) they included patient information within the scope of emergency care, with internal medical issues as the chief complaints; (ii) ML was utilized for making predictions, and the performance of the employed ML model was assessed using evaluation metrics; (iii) the prediction outcome involved ED URVs; (iv) the study report clearly defined the interval between a patient’s first and return visits; and (v) the study report provided a clear description of the scale of its analyzed patient population.

By contrast, articles with the following features were excluded from this study: (i) not written in English; (ii) related to traumatic diagnoses; (iii) not using ML methods for prediction; and (iv) the prediction of other outcomes—such as mortality, intensive care unit admission, and ED length of stay—without URV prediction.

### Study selection and data extraction

Two reviewers independently screened the titles and abstracts of the articles found and conducted a full-text review of each to determine its eligibility for inclusion in this study. To increase efficiency, we divided the study selection and data extraction processes into blocks based on the publication date, beginning with articles published between 2010 and the search date of February 2023. All acceptable articles were then screened independently by the same two reviewers in full-text form, with each reviewer kept unaware of the other’s findings. Any conflicts that arose during study selection or data extraction process were resolved through discussion and consultation with a third member of the research team.

### Outcome measures and data synthesis

The primary outcomes of this study were the determination of the efficacy of each analyzed ML model in predicting ED return visits and the determination of the proportion of methods used among the selected articles. We employed descriptive statistics to summarize the characteristics of the studies included in our analysis. Furthermore, the predictive performance of each ML model was analyzed and reported on the basis of the interval of return visits, patient population, and research scale.

## Results

### Study characteristics

**Fig. 1:**
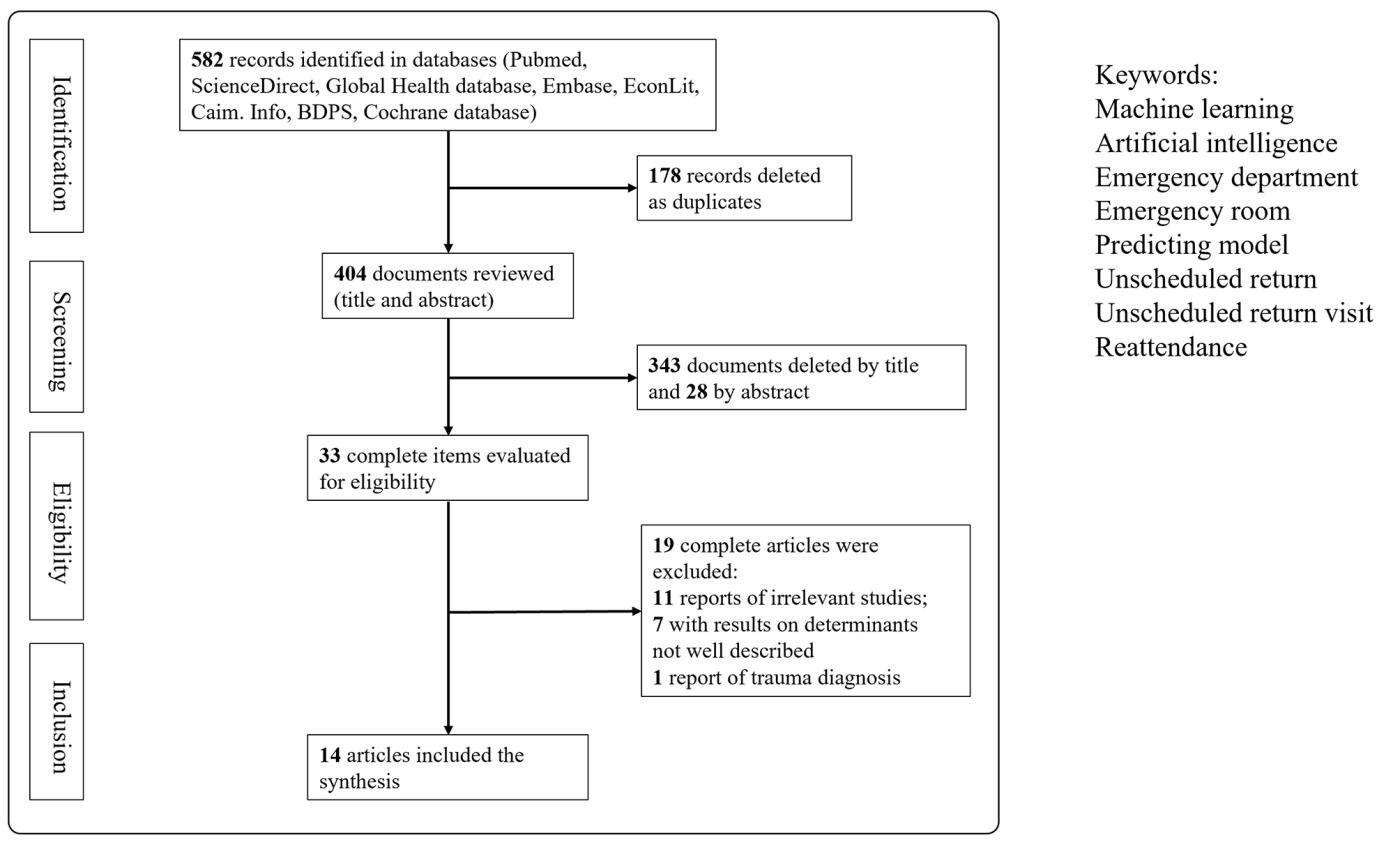
PRISMA flow diagram of the study selection process

Of the 582 articles identified through our electronic search, 33 were selected for full-text review. Finally, 14 articles published between 2010 and 2022 were analyzed in detail, as shown in Fig. 1 and summarized in Table 1. These 14 studies were conducted in multiple countries, namely seven in the United States, three in Taiwan, two in Singapore, one in the United Kingdom, and one in Portugal.

**Table 1:** Characteristics of the selected articles

Study, Year	Country	Research scale	Subject group	Number	Record period	ML model	Predictive power	Validation
**72-hour URVs**								
Lee et al. 2012	US	Single center	Pediatric patients	66,861	2009	DAMIP model	AUC 0.85	AUC 0.831
Hu et al. 2017	Taiwan	Nation-based	Pediatric patients	125,940	1998 to 2009	Naïve Bayes model	AUC 0.644	x
						Simple Cart model	AUC 0.721	x
						RF model	AUC 0.723	x
						LR model	AUC 0.718	x
Pellerin et al. 2018	US	Multicenter (4)	All patients	21,141	2014	LR model	AUC 0.74	AUC 0.7279
Fernades et al. 2019	Portugal	Single center	Adult patients	511,301	2012 to 2016	LR model	AUC 0.842	x
						SVM model	AUC 0.791	x
Meng et al. 2019	Singapore	Single center	All patients	328,733	2011 to 2013	LR model	Accuracy 0.67	AUC 0.66
						DAMIP model	Accuracy 0.728	AUC 0.728
Hong et al. 2019	US	Multicenter (2)	Adult patients	330,631	March 2013 to July 2017	LR model	72 h-URV: AUC 0.69	AUC 0.692
							9d-URV: AUC 0.71	AUC 0.708
						XGB model	72 h-URV: AUC 0.73	AUC 0.717
							9d-URV: AUC 0.73	AUC 0.727
Chen et al. 2021	Taiwan	Nation-based	Patients older than 65	49,252	1996 to 2010	Decision tree-based model	AUC 0.768	x
Chimel et al., 2021	UK	Single center	Adult patients	44,294	April 2019 to April 2020	XGB model	AUC 0.761	AUC 0.747
Hsu et al. 2022	Taiwan	Multicenter (2)	Patients with abdominal pain	290,914	2018 to 2019	LR model	AUC 0.73	x
						RF model	AUC 0.71	x
						XGB model	AUC 0.74	x
						Reduced VC model	AUC 0.72	x
Xie et al. 2022	Singapore	Nation-based	All patients	216,877	2011 to 2019	LR model	AUC 0.683	x
						RF model	AUC 0.666	x
						XGB model	AUC 0.700	x
						AutoScore model	AUC 0.673	x
						MLP model	AUC 0.696	x
						Med2Vec model	AUC 0.673	x
						LSTM model	AUC 0.694	x
**30-day URVs**								
Hao et al. 2014	US	Nation-based	All patients	293,461	2012	Decision tree-based model	AUC 0.71	x
Suffoletto et al. 2016	US	Multicenter (2)	Patients older than 65	202	2015	LR model	AUC 0.69	x
Poole et al. 2016	US	County-based	All patients	1,125,118	2010 to 2014	LR model	AUC: 0.77	x
						Lasso model	AUC 0.84	x
						RF model	AUC 0.98	x
Fowler et al. 2017	US	Single center	All patients	91,297	2010 to 2015	XGB model	AUC 0.75	x

URVs: unscheduled return visits; ML: machine learning; AUC: area under the curve; DAMIP: Discriminant Analysis Via Mixed Integer Programming; RF: Random Forest; LR: Logistic Regression; SVM: Support Vector Machine; XGB: Extreme Gradient Boosting; VC: voting classifier; MLP: Multilayer perceptron; LSTM: Long short-term memory; US: United States; UK: United Kingdom

The selected studies analyzed the interval between each patient’s first visit and return visit; these intervals were then divided into groups of 72 h, 9 days, and 1 month. Nine articles exclusively predicted 72-hour return visits; Hong et al. predicted two outcomes, namely return visits within 72 h and return visits within 9 days [17]; and four articles predicted return visits within 30 days.

### Data sample and predictors

In all the 14 selected articles, the study population comprised patients visiting EDs, with sample sizes approximately ranging from 200 to 1.25 million individuals. The study conducted by Suffoletto et al. [12] had the fewest participants; that study focused on patients older than 65 years, was conducted in two hospitals with 404 and 520 beds, and analyzed only 202 participants. By contrast, Poole et al. used the dataset of the Indiana Public Health Emergency Surveillance System, which covers more than 1.25 million patients and contains medical data from multiple institutions [11]. Although the data used for ML model implementation were specific to each study, several common categories were identified. These categories included demographic variables (e.g., age, sex), clinical variables (e.g., vital signs, diagnoses based on *International Classification of Diseases* codes), arrival information (e.g., arrival time, triage level, transport mode), and types of examinations (e.g., blood tests, images). Ten of the 14 analyzed articles included information regarding comorbidities or medical history [3, 9, 11, 12, 15, 17–21]. In addition, six of the articles considered chief or triage complaints, with those without chief complaints as variables using diagnoses instead [12–14, 16, 20, 21]. Four studies presented information regarding the use of hospital metrics (e.g., number of prior ED visits, number of prior hospitalizations) [3, 14, 19, 20]. Finally, in addition to using clinical variables, five articles linked their data to paramedical information, such as ethnicity, socioeconomic status, educational level, and insurance status [3, 10, 11, 20, 21].

### ML process

#### Candidate variable handling and feature engineering

In most of the selected studies, all the variables were included in the implemented models. Both Fernandes et al. [16] and Poole et al. [11] used stepwise methods for feature selection to reduce the number of input variables.

#### Data resampling

In most of the selected articles, the datasets were randomly divided into training and testing datasets. Cross-validation, which can help prevent the overfitting or underfitting of a model, was used in five of the selected studies [3, 9, 15, 17, 19].

#### Prediction algorithms and calibration of parameters

**Fig. 2:**
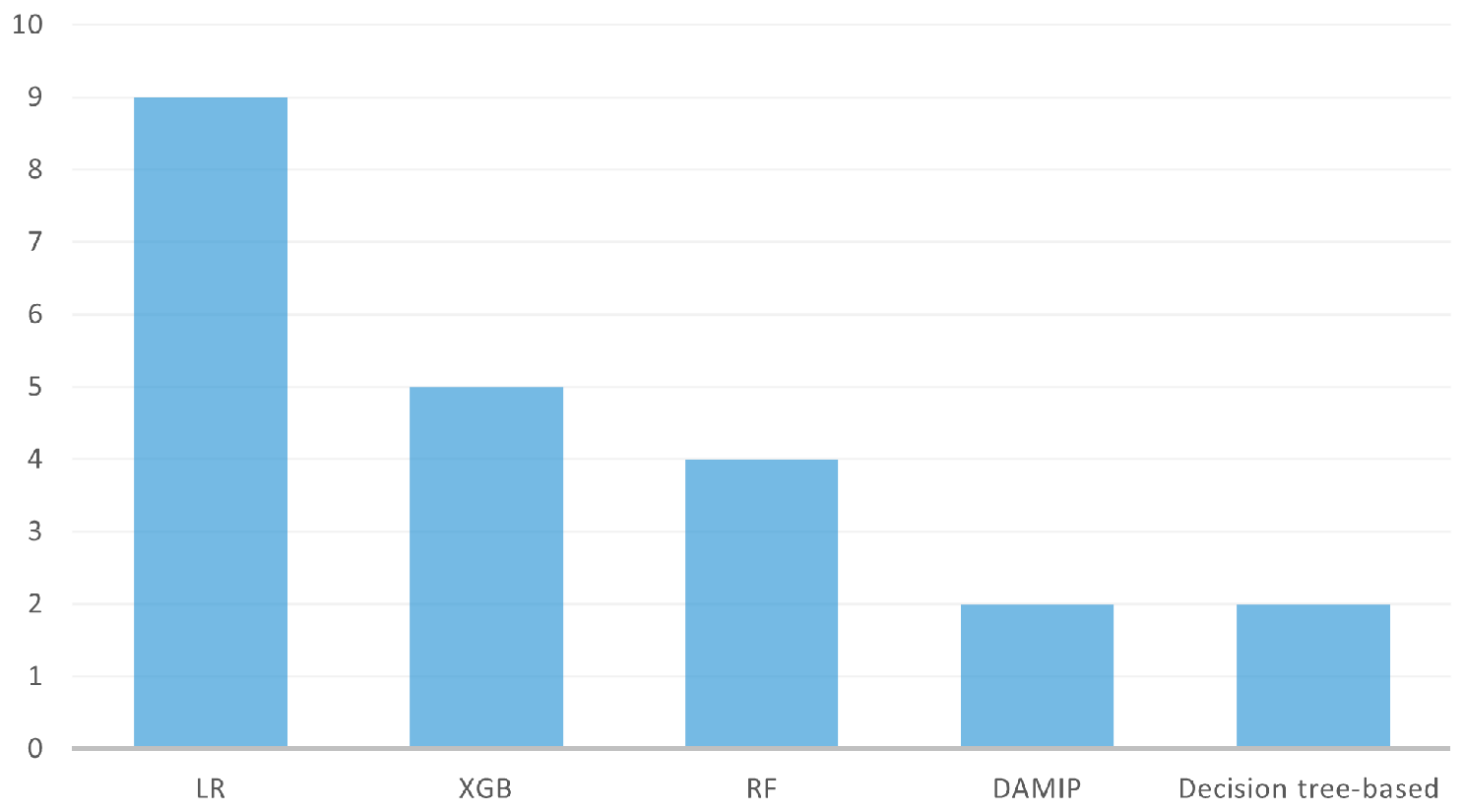
Frequency of commonly used ML models in the included studies

In total, 33 models were used to predict ED return visits. Logistic regression (LR; *n* = 9/14 articles) and eXtreme Gradient Boosting (XGB; *n* = 5/14) were the two most widely used methods, followed by random forest (RF; *n* = 3/14) and then discriminant analysis using mixed integer programming (DAMIP; *n* = 2/14) and decision tree–based models (*n* = 2/14) (Fig. 2). Some models were used in only one study. Only nine models in five studies used the cross-validation method to validate the model performance or to tune hyperparameters. R and Python were the most commonly used tools.

#### Evaluation metrics

The metrics used to evaluate the performances of the tested models included the area under the receiver operating characteristic (ROC) curve (AUC), sensitivity and specificity, and accuracy. The ROC-AUC metrics were the most frequently used.

#### Model agnostic methods

The majority of the authors used LR coefficients to determine significant variables. Feature importance analysis was implemented in seven studies to calculate the importance of the predictors [10, 11, 13, 17, 19–21].

### Model performance assessment

#### Interval between two visits (72 h, 9 days, 30 days)

**Fig. 3:**
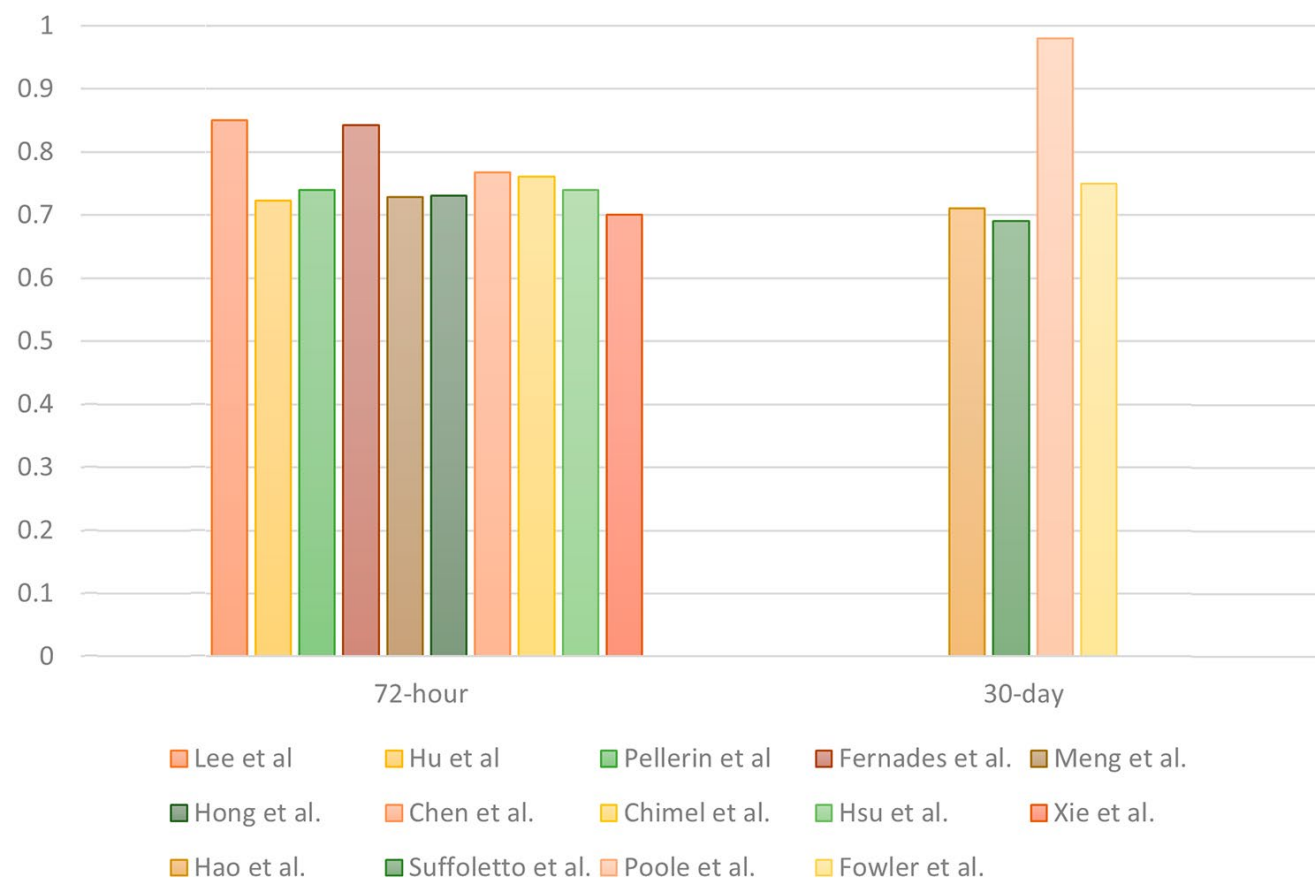
Highest AUCs in all the analyzed studies

Most of the studies (*n* = 11/14) focused on predicting URVs within a 72-hour interval. A total of 25 models were developed to predict 72-hour URVs, whereas two models were developed for 9-day URVs and six were developed for 30-day URVs. The AUC was used to evaluate the performances of the predictive models; the corresponding results are presented in Table 1. For 72-hour URVs, LR was the most commonly adopted method, with a median AUC of 0.72 and an interquartile range (IQR) of 0.69–0.77. XGB exhibited similar predictive power across all the studies where it was used, with a median AUC of 0.73 and an IQR of 0.71–0.76. For 30-day URVs, the AUC ranged from 0.69 to 0.98, with LR achieving the lowest score of 0.69 in the study with 202 patients [12]. The RF model achieved the highest score, namely 0.98 in the study with approximately 1.25 million patients [11]. The highest AUCs of all the studies are presented in Fig. 3.

#### Patient population

**Fig. 4:**
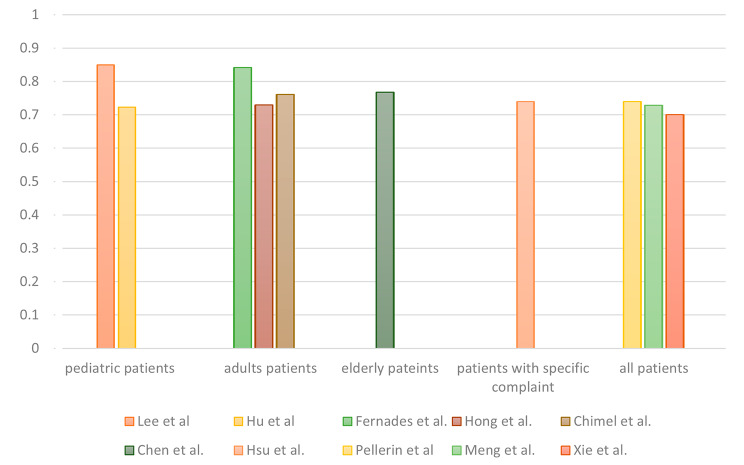
Highest AUCs for 72-hour URVs determined by the predictive models for multiple patient groups

Different target groups were present in the 14 analyzed studies. Six articles analyzed all patients who visited the included EDs, whereas others focused on specific age groups, including adult patients (*n* = 3/14), pediatric patients (*n* = 2/14) and older adult patients (*n* = 2/14). Hsu et al. conducted the only study that focused on adult patients with abdominal pain [20]. For 72-hour URV prediction across the various patient populations, the AUCs ranged from 0.7 to 0.85, and no significant differences between target groups were observed (Fig. 4).

#### Research scale

**Fig. 5:**
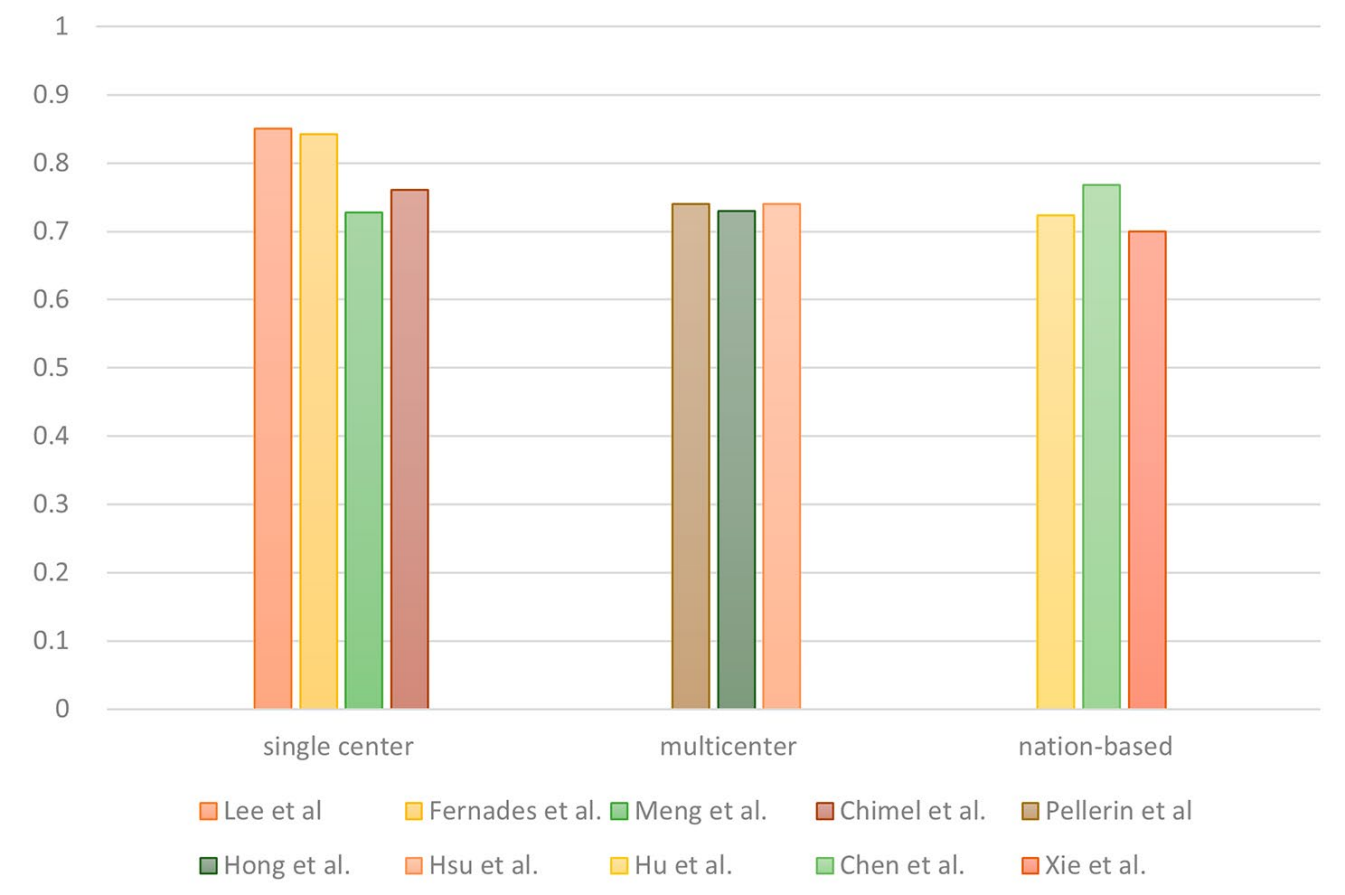
Highest AUCs for 72-hour URVs determined by the predictive models for multiple research scales

The selected articles were divided into single-center, multicenter, and national database studies on the basis of their research scale. Five of the articles used data from a single hospital, and four articles analyzed data from multiple hospitals. In addition, five articles employed national or statewide databases from the United States, Taiwan, Singapore, or the American state of Indiana. For 72-hour URVs, no evident differences in AUCs were observed among studies with different research scales (Fig. 5).

## Discussion

To the best of our knowledge, the present study was the first to attempt to provide a comprehensive overview of the use of ML models for predicting URVs among patients who presented to EDs. Our study discusses the application of ML-based decision support systems for predicting the probability of URVs to EDs based on existing literature. Regarding the ED revisit intervals, the initial timeframe we selected for inclusion was within 1 month, a cutoff borrowed from Medicare’s Hospital Readmission Reduction Program [26]. Although periods ranging up to 30 days have been used as timeframes for ED return visits in previous studies, a shorter timeframe of 72 h may be more useful in identifying return visits related to the previous episode of care and also identifying preventable revisits [27]. In our analysis, no evident differences in prediction accuracy between different timeframes were observed among the selected studies, with the exception of the country-based study conducted by Poole et al., which aimed to predict ED revisits within 1 to 6 months [11]. Developing ML models to predict 72-hour URVs to an acceptable degree of accuracy is feasible according to our review given that 72-hour URVs, rather than 30-day URVs or longer-term URVs, are more relevant to quality of care in EDs.

According to our results, LR was the most widely utilized method; however, it may not consistently yield the highest predictive accuracy compared with other models, even after undergoing multiple corrections and adjustments. In addition, linear relationships may not exist between variables; thus, a nonlinear model is necessary. RF has been frequently employed in related studies because it is a robust classifier. XGB has also been used extensively because of its ability perform well with imbalanced data by autoregulating class weights during training [28]. Among the commonly used models, XGB usually demonstrates the best predictive ability [29]; however, XGB has some shortcomings, such as false-negative preference [20]. An ensemble model, like voting classifier, combining multiple models with a specific weighting ratio has been adopted in some studies to achieve relatively favorable balance [16, 20, 21].

Overall, although some studies have achieved high predictive accuracy, improving the accuracy of predicting ED URVs to beyond 0.75 appears to be challenging. A primary limitation is that many studies record only reattendance without describing reasons for such reattendance or subsequent outcomes. Such a lack of differentiation can make distinguishing between patients with conditions with varying levels of severity difficult and ultimately can hinder the accuracy of prediction [30, 31]. URVs can be related to diseases, physicians, or patients. URVs caused by physician- or disease-related problems may be predicted by certain metrics; however, repeated ED attendance for patient-related reasons—such as free medical consultation cards, psychiatric problems, and social problems—is relatively difficult to predict given the complexity of such reasons, including health status and behavior. Most related studies have used only quantitative data, such as that related to lab values and vital signs, and have overlooked other types of data, such as imaging and text-based records, psychiatric and behavioral problems, and socioeconomic status. Such data may be difficult to extract from electronic health records; nevertheless, the accuracy of predictive models may be limited without the information [16, 18]. Additionally, most data used in ML models are collected only from the hospitals that patients visited first and do not include records from other nearby medical institutions; this drawback can result in incomplete patient histories or data [17, 27]. For example, patients may not return to the same hospital after their first visit because the accessibility of different hospitals in some country is high [20].

The models presented by most of the studies analyzed in the present study had similar predictive accuracies, and three of the studies had a higher-than-average accuracy [9, 11, 16]. Lee et al. conducted their study in a pediatric ED with a relatively low daily visit count and a relatively uniform patient population with few underlying conditions. The DAMIP approach used in their study established a classification rule based on a training set, which helped achieve higher blind predictive accuracy with small numbers of variables and an independent sample of patients [9, 15].

Poole et al. achieved exceptionally high predictive accuracy by using the RF model [32]. That study used a dataset that covered more than 1.25 million patients and medical data from multiple institutions and thus provided comprehensive patient histories. Additionally, those researchers focused on predicting URVs at the patient level rather than at each encounter, which allowed for more accurate prediction of the overall revisit risk [11, 13].

Compared with other studies that have used only quantifiable data, the study conducted by Fernandes et al. demonstrated a significant increase in accuracy when both textual and numerical data were employed for prediction. However, the precision of their model was low, which led to a relatively high false-positive rate [16]. In summary, data extraction from multiple nearby medical institutions and the inclusion of both textual and numerical data may increase the accuracy of ML models in predicting ED URVs; however, some conditions limit their practicality and range of applicability.

Previous studies have indicated that older age is associated with a higher risk of ED reattendance, and the presence of atypical or nonspecific geriatric syndromes may decrease the accuracy of return visit prediction [33]. Conversely, pediatric patients tend to have simpler medical histories, leading to higher predictive accuracy [15]. However, our review found no significant differences in predictive power among the patient subgroups of older adult patients, pediatric patients, adult patients, and all patients between the selected studies. Even when specific chief complaints were considered, predictive accuracy remained similar [20]. With regard to research scale, some studies have suggested that the results of single-center and multicenter studies may not fully capture return visits to EDs in other hospital systems, potentially leading to the underestimation of ED URVs [3, 5, 17]. Accordingly, in the present study, no evident superiority in predictive accuracy was noted for the national-level or multicenter research scales; however, this comparison was imprecise because many differences in conditions between the selected studies were present.

While developing a practical ML model for predicting ED URVs to a reasonable degree of accuracy appears feasible, it’s important to acknowledge that achieving this accuracy relies on the integration of multiple data sources and dimensions, as indicated by previous research [30, 34]. However, integrating detailed data within a limited timeframe could be a challenging task [27, 31]. In addition, although models that predict ED URVs improve patient safety and reduce costs, these models may have limited efficacy when applied in the real world. Thus, further empirical research is necessary.

### Limitations

This study had some limitations. First, this study was a scoping review study, and thus although the inclusion of qualitative content enabled contextualization of the current ML evidence base, related quantitative outcomes may have been inadequate. Second, ML techniques and skills are evolving rapidly, and thus the conditions governing ML models, as well as their performances, could change quickly. Finally, the present study selected only articles published in English, and thus some key information published in another language or other languages may have been overlooked.

## Conclusion

To the best of our knowledge, this study is the first to summarize the use of ML models for predicting URVs in ED patients. The results indicated the feasibility of developing practical ML models for predicting ED URVs. LR was the most widely utilized method, whereas XGB generally demonstrated the highest predictive ability. In addition, this study found that including multiple data sources and dimensions is vital for enabling ML models to achieve higher accuracy but that such inclusion could be challenging within a limited timeframe. Finally, the application of ML models for predicting ED URVs may improve patient safety and reduce medical costs; however, further research is necessary to explore the real-world effects of such application.

## Data Availability

All data used and analyzed during this study are included in this published article.

## Abbreviations

URV: Unscheduled return visit
ED: Emergency department
ML: Machine learning
PRISMA: Preferred reporting items for systematic review and meta-analysis
LR: Logistic regression
XGB: eXtreme Gradient Boosting
RF: Random forest
DAMIP: Discriminant analysis using mixed integer programming
ROC: Receiver operating characteristic
AUC: Area under the curve
